# Synthesis of Pure Enantiomers of Titanium(IV) Complexes with Chiral Diaminobis(phenolato) Ligands and Their Biological Reactivity

**DOI:** 10.1038/s41598-018-27735-0

**Published:** 2018-06-26

**Authors:** Maya Miller, Edit Y. Tshuva

**Affiliations:** Institute of Chemistry, The Hebrew University of Jerusalem, Jerusalem, 9190401 Israel

## Abstract

Racemic and enantiomerically pure titanium(IV) complexes with *ortho*-brominated or *para*-nitrated chiral diaminobis(phenolato) ligands were prepared with NH and NMe cyclohexyldiamino bridges through ligand to metal chiral induction. The hydrolytic behavior of the complexes was evaluated, identifying the N-methylated complex as the most stable. A representative NH complex hydrolyzed to first give a dimeric structure in solution as deduced by NMR diffusion measurements, followed by formation of clusters with higher nuclearity, as was supported by X-ray characterization of a tetranuclear cluster obtained in trace amounts following 30 days in water solutions. The cytotoxicity of the enantiomerically pure and racemic complexes was measured on HT-29 human colon cancer cell line based on the MTT assay; all stereochemical configurations of the N-methylated complex were inactive, whereas for the NH complexes, the racemic mixtures were mostly inactive but the pure enantiomers exhibited similarly high cytotoxicity, supporting a polynuclear active species. Analysis of the two enantiomers of the most active brominated complex for their cytotoxicity on human ovarian A2780, cisplatin resistant A2780cp and multi-drug-resistant A2780adr cell lines as well as for their apoptosis induction on the A2780 line revealed similar reactivity, supporting a similar mechanism for the two enantiomers.

## Introduction

**Figure 1:**
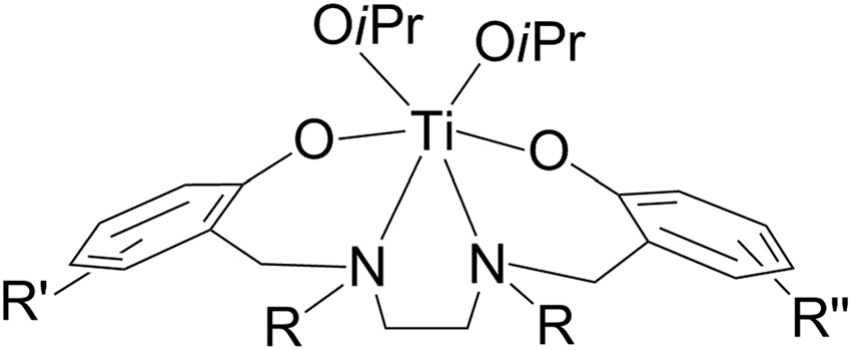
Salan titanium(IV) complexes.

Titanium(IV) compounds were the first non-platinum based metallodrugs entering clinical trials for treatment of cancer^1–7^. Two classes of compounds based on cyclopentadienide and diketonato ligands exhibited a wide range of activity and mild toxicity *in vivo*^8–21^, but their rapid hydrolysis under biological environment to form multiple unidentified products hampered further development^14,22,23^. Later “salan” type diaminobis(phenolato) Ti(IV) compounds (Fig. 1) showed: (a) markedly improved hydrolytic stability eventually giving defined polynuclear hydrolysis products, and (b) a wide range of activity both *in vitro* and *in vivo*, with no sign of toxicity to treated animals^13,24–34^. Wide structure-activity relationship studies pointed to a negative effect of steric bulk and a positive effect of *ortho*-halogenation on hydrolytic stability and cytotoxicity.

The salan type Ti(IV) complexes are chiral, exhibiting *C*_2_ or *C*_1_ symmetry. Therefore, when considered for medicinal applications, the evaluation of the biological activity of the pure enantiomers as well as their racemic mixture is essential^35,36^. In previous studies, employing chiral *trans*-cyclohexyldiamine- and bipyrrolidne-based ligands induced ligand-to-metal chiral induction affording enantiomercally pure compounds that were analyzed for cytoxicity^37–40^. In general, the enantiomerically pure forms of cyclohexyl-based NH complexes gave higher cytotoxicity relative to that of the racemic mixture^37^; however, for related active N-Me complexes, the racemic mixture was generally more active than the pure enantiomers^38^. In contrast, for the bipyrrolidine-based complexes, the racemate was inactive whereas its enantiomerically pure isomers had similar biological activity^40^. Following studies suggested that the hydrolysis products participate as the active specie inside the cell^41–44^, providing an explanation to the different reactivity of racemates relative to that of the enantiomerically pure forms; whereas pure enantiomers gave homochiral dimeric clusters, the racemic mixtures produced heterochiral diastereomeric dimers^37,40^. This paper presents structure-activity relationship studies of differently substituted cyclohexyl-based complexes, analyzed as enantiomerically pure and as racemic. Preliminary mechanistic studies suggest a similar mode of action for the two active enantiomers.

## Results and Discussion

### Synthesis and Characterization

**Figure 2:**
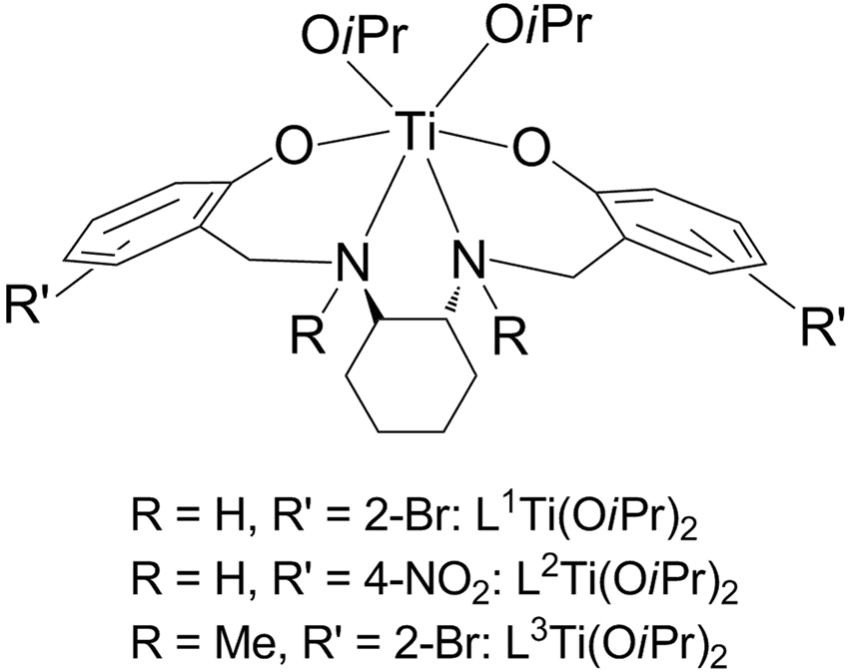
Titanium(IV) complexes of chiral cyclohexyldiaminobis(phenolato) ligands.

Three sets of salan cyclohexyldiaminobis(phenolato) Ti(IV) complexes were synthesized according to a published procedure; each was produced as two separate enantiomers (Δ or Λ at metal) through ligand-to-metal chiral induction from optically pure *trans*-1,2-diaminocyclohexane (*R,R* or *S,S* at ligand, respectively)^45–47^, and as a racemic mixture (Δ + Λ at metal) starting from the racemic form of the starting material (Fig. 2). The compounds differed in the aromatic substitution and the diamino bridge. *Ortho*-bromination was selected for enhancing hydrolytic stability as reported previously^26,32–34,40^, and *para*-nitration was selected for improving solubility^26,34^.

Ligands L^1–3^H_2_ were synthesized according to a published procedures^37–39,48^; ligand L^1,2^H_2_ were obatined by a condensation reaction between the substituted benzaldehyde and *trans*-diaminocyclohexane, and L^3^H_2_ was obtained by methylation of L^1^H_2_ with formaldehyde. The ligands were characterized by NMR and optical rotation. The corresponding Ti(IV) complexes were produced by reacting L^1–3^H_2_ with Ti(O*i*Pr)_4_ in THF, at room temperature, for 12–72 hours^37–39^. ^1^H NMR confirmed that the desired complex had been obtained quantitatively in >95% optical purity.

**Figure 3:**
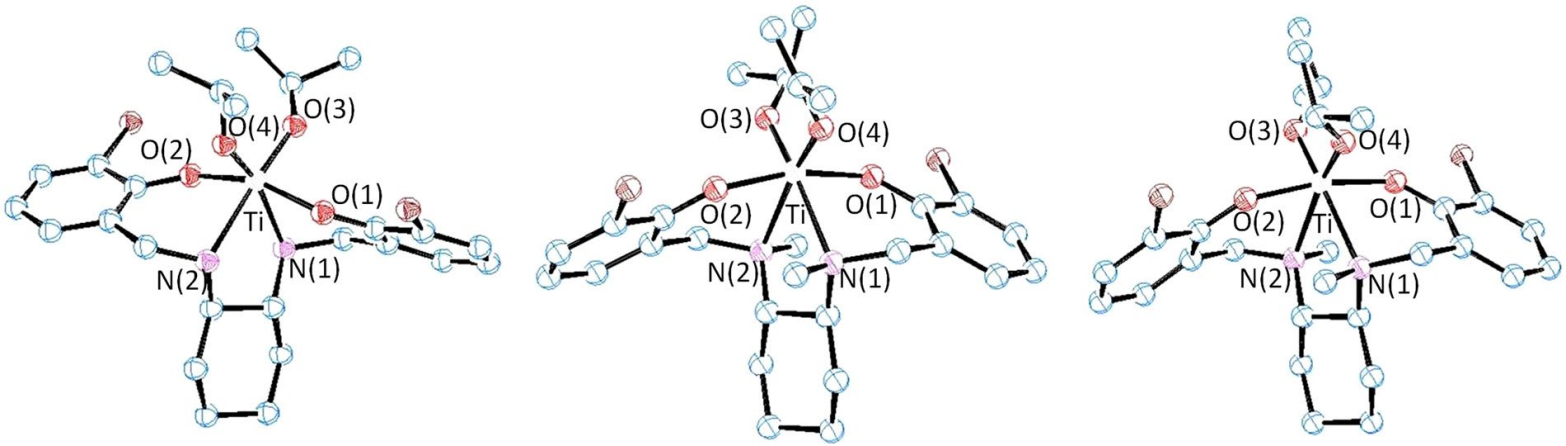
ORTEP presentations of *rac*-L^1^Ti(O*i*Pr)_2_ (left), *rac*-L^3^Ti(O*i*Pr)_2_ (middle) and *S,S*,Λ-L^3^Ti(O*i*Pr)_2_ (right) with 50% probability ellipsoids; H atoms were omitted for clarity. Selected bond lengths [Å] and angles [°] for the three compounds respectively: Ti-O(1) 1.950(2), 1.930(4), 1.914(2), Ti-O(3) 1.791(2), 1.800(4), 1.817(2), Ti-N(1) 2.250(2), 2.324(4), 2.360(2), O(1)-Ti-O(2) 159.56(8), 165.95(2), 169.20(7), O(3)-Ti-O(4) 105.92(1), 106.77(2), 106.78(8), N(1)-Ti-N(2) 75.31(8), 74.42(2), 73.94(7).

Single crystals suitable for X-ray crystallography were obtained at −30 °C for three compounds: *rac*-L^1^Ti(O*i*Pr)_2_ crystallized from diethyl ether, and *rac*-L^3^Ti(O*i*Pr)_2_ and *S,S*,Λ-L^3^Ti(O*i*Pr)_2_ crystallized from a mixture of hexane and dichlormethane, the latter confirmed the aforementioned chiral induction. The structures are presented in Fig. 3 (Supplementary Table S1). All structures featured a *C*_2_ symmetry, similarly to previously reported related Ti(IV) salan compounds^37–40^. The Ti(IV) center exhibited an octahedral geometry, whereby the phenolato oxygen atoms were in a *trans* configuration and the isopropoxo groups were in a *cis* orientation.

### Hydrolysis

The comparative hydrolytic stability was assessed using ^1^H NMR as previously described^25,26^, adding 10% D_2_O (>1000 equivalents) to THF-*d*_8_ solutions of the compounds and monitoring the signals corresponding to the *iso*-propoxo labile ligands overtime. All experiments were conducted on the *S,S*,Λ- stereoisomers. The ${{\rm{t}}}_{\frac{1}{2}}$ values are presented in Table 1.

**Table 1:** ${{\rm{t}}}_{\frac{1}{2}}$ values for hydrolysis of the labile ligands of L^1–3^Ti(O*i*Pr)_2_ at 1:9 D_2_O/THF-*d*_8_ solution at room temperature based on pseudo first order fit and relative IC_50_ [*μ*M] values for the complexes and cisplatin toward human colon HT-29 cancer cell line (standard deviation of means were calculated for at least 3 repeats); ^a^measured on the *S,S*,Λ-stereoisomer.

Complex	${{\rm{t}}}_{\frac{1}{2}}$^a^ [hr]	IC_50_ [*μ*M]		
		*Racemic*	*R,R*,Δ	*S,S*,Λ
L^1^Ti(O*i*Pr)_2_	4	Inactive	4.1 ± 0.8	4.9 ± 1.4
L^2^Ti(O*i*Pr)_2_	14	Inactive	4.2 ± 1.8	4.6 ± 1.5
L^3^Ti(O*i*Pr)_2_	385	Inactive	Inactive	Inactive
Cisplatin	—	11.1 ± 0.4^56^ [*μ*M]		

In agreement with previous studies^32,37,38,40^, the most hydrolytically stable compound was the N-methylated complex L^3^Ti(O*i*Pr)_2_. Comparing the stability of the two NH compounds, similar stability, or even slightly higher stability for the nitrated compound implied that the *ortho*-bromination or the electron withdrawal by the nitro groups had a smaller effect on stability relative to that of the secondary amine, whereby added stability was provided by the cyclohexyl group relative to that of the ethylenediamino-based counterparts previously described^26,32,37–40^. Inspecting the ^1^H NMR of the product of the hydrolysis of *S,S*,Λ-L^1^Ti(O*i*Pr)_2_, six aromatic signals evinced that a product of high symmetry was obtained, presumably dimeric^37,40^. The more complex corresponding spectrum of the hydrolysis product of the racemic complex implied that a mixture of homo- and hetero-chiral clusters had been obtained (Supplementary Figs. S1 and S2)^37,40^.

**Figure 4:**
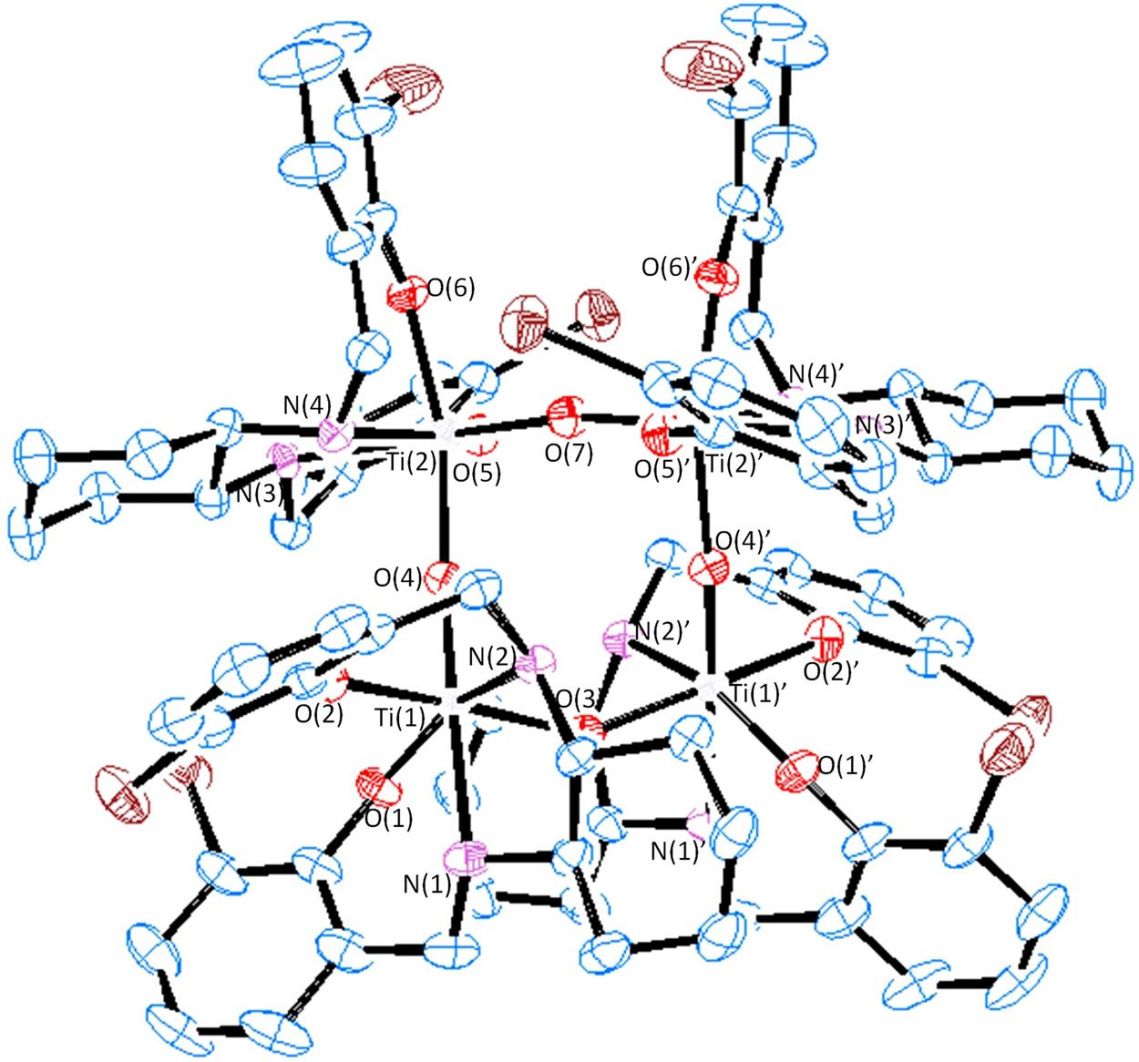
ORTEP presentation of Ti_4_(*μ*-O)_4_(*S,S*-L^1^)_4_ at 50% probability ellipsoids; H atoms and solvent were omitted for clarity. Selected bond lengths [Å] and angles [°]: Ti(2)-O(7) 1.820(1), Ti(2)-O(6) 1.911(3), Ti(2)-N(3) 2.294(4), O(7)-Ti(2)-O(4) 92.7(2), O(5)-Ti(2)-O(6) 94.1(2), N(3)-Ti(2)-N(4) 74.4(2).

To gain more structural information on the hydrolysis product of L^1^Ti(O*i*Pr)_2_, the compound (*S,S*,Λ-stereoisomer) was reacted with water (>10,000 equivalents) for 30 days and the product was re-dissolved in diethyl ether and allowed to crystallize. Single crystals suitable for X-ray crystallography were obtained in trace amounts, and the structure is depicted in Fig. 4 (Supplementary Table S2). The structure (R $\overline{3}$ *c* space group) features a tetrameric species of the type Ti_4_(*μ*-O)_4_(*S,S*-L^1^)_4_ with four titanium centers bridged by oxo atoms, each metalo center binding a salan ligand with the phenolato donors having shifted to a *cis* orientation. The bond lengths and angles were generally similar to those of known related compounds^37,43,49,50^. As this structure should yield twelve aromatic signals in the ^1^H NMR due to its *C*_2_ symmetry, it is evident that the tetrameric complex is not the main product obtained in the hydrolysis reaction described above.

To shed more light on the possible hydrolysis products obtained in solution, diffusion NMR measurements were applied on *S,S*,Λ-L^1^Ti(O*i*Pr)_2_ upon addition of water using diffusion order spectroscopy (DOSY), whereby the diffusion coefficient (D) derived is proportional to the compound size (Table 2, Supplementary Fig. S3)^51–53^. Within 24 hours from water addition, the dominant species was a dinuclear compound, in agreement with previous studies on related salan diaminocyclohexyl-based complexes^37,40^ and in correlation with the aforementioned ^1^H NMR spectrum of the hydrolysis reaction. Prolonged incubation in water solutions gave indication of further decomposition to give products of higher nuclearity in trace amounts. It is plausible that the dimer forming following water replacement of the isopropoxo ligands further reacts in an associative mechanism as previously suggested, to give the tetranuclear species as implied by the *cis* configuration of the phenolato units^25^.

**Table 2:** Diffusion coefficients (D) values [10^−10^ m^2^/s] for *S,S*,Λ-L^1^Ti(O*i*Pr)_2_ before and after addition of water; ^a^the main product (D = 4.436 10^−10^ m^2^/s) was accompanied by another product, presumably trimeric, in trace amounts.

Time [h]	D [10^−10^ m^2^/s]
0	5.572
24	4.436
48	4.436; 4.335^a^

### Cytotoxicity

**Figure 5:**
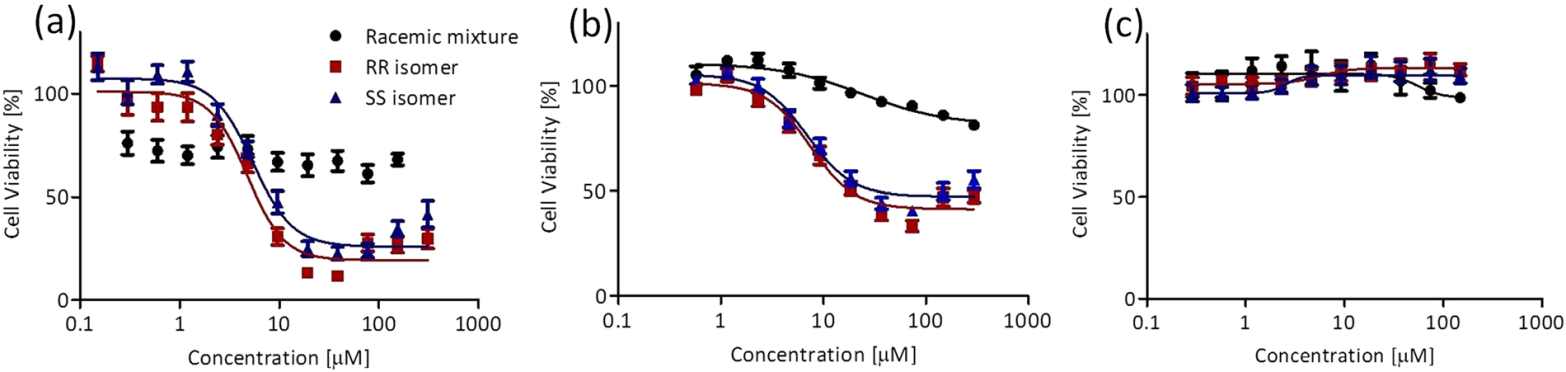
Dependence of HT-29 cell viability based on the MTT assay following a three days incubation period on added concentration of L^1^Ti(O*i*Pr)_2_ (**a**); L^2^Ti(O*i*Pr)_2_ (**b**) and L^3^Ti(O*i*Pr)_2_ (**c**) presented on a logarithmic scale. Standard error of means were determined by a nonlinear regression of a variable slope (four parameters) model by Graph Pad Prism5.0 program for at least 9 repeats.

The anti-proliferative activity of the compounds was evaluated on human colon HT-29 cancer cells by the MTT (methylthiazolyldiphenyl-tetrazolium) assay as previously described^54^. The results are illustrated in Fig. 5 and a summary of the relative IC_50_ values is provided in Table 1.

Inspecting the reactivity of L^1,2^Ti(O*i*Pr)_2_, both with NH donors, no biological activity was detected for *rac*-LTi(O*i*Pr)_2_^37^, whereas for each, the enantiomerically pure *R,R*,Δ- and *S,S*,Λ-LTi(O*i*Pr)_2_ had similarly high cytotoxicity with no significant difference, in accordance with previous observations^40^. Although the nitration was presumed to increase solubility as is also manifasted by increased activity relative to other *para*-substituted derivatives^26,34,37^, the maximal inhibition obtained by L^2^Ti(O*i*Pr)_2_ is slightly lower. Inspecting the reactivity of L^1,3^Ti(O*i*Pr)_2_, both with *o*-Br, it is evident that the N-methylation reduced the activity also for the enantiomerically pure forms^38,40^. This observation may be explained by the added steric influence of the N-Methylated cyclohexyl ring together with the steric aromatic substitutions, overall creating sufficient bulk to abolish the activity. Similar effect was obtained for complexes with non chiral ligands as previously described^32^.

**Figure 6:**
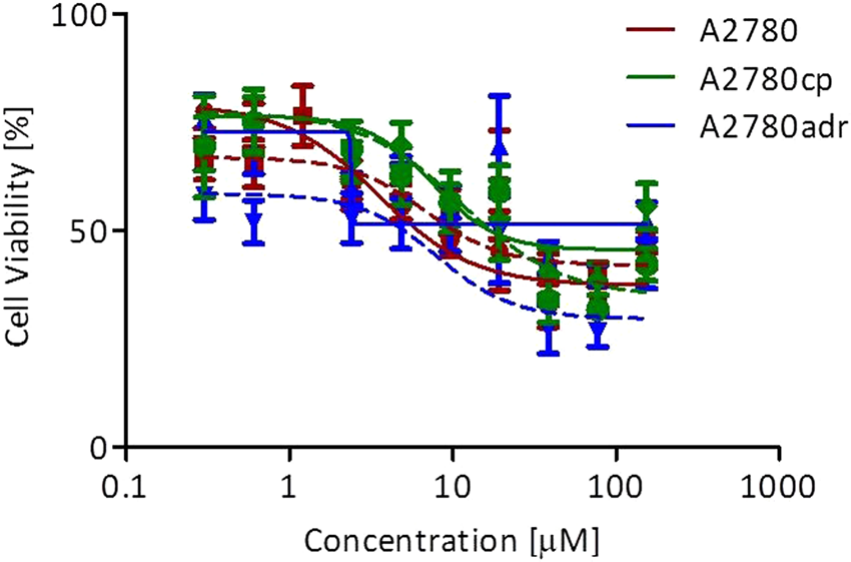
Dependence of ovarian carcinoma cancer cell lines (A2780: red, A2780cp: green, A2780adr: blue) cell viability based on the MTT assay following a three days incubation period on added concentration of *R,R*,Δ- (continuous line)/*S,S*,Λ- (dashed line)L^1^Ti(O*i*Pr)_2_ presented on a logarithmic scale. Standard error of means were determined by a nonlinear regression of a variable slope (four parameters) model by Graph Pad Prism5.0 program for at least 9 repeats.

The most active L^1^Ti(O*i*Pr)_2_ was selected for further studies, aiming to investigate possible differences between the two enantiomers. Thus, cell viability studies were performed for the two enantiomers based on the MTT assay on human ovarian A2780 and its resistance lines: cisplatin-resistant human ovarian A2780cp and multi-drug-resistant (MDR) human ovarian A2780adr. The results are illustrated in Fig. 6 and a summary of the relative IC_50_ values is provided in Table 3. Marked activity was obtained for all lines tested; importantly, similar activity was recorded for the two enantiomers on all lines, implying that there are no stereospecific interactions that are essential for overpassing drug resistance.

**Table 3:** Relative IC_50_ [*μ*M] values for complexes *R,R*,Δ/*S,S*,Λ-L^1^T*i*(O*i*Pr)_2_ toward human ovarian carcinoma cancer cell lines (standard deviation of means were calculated for at least 3 repeats).

IC_50_ [*μ*M]		
	*R,R*,Δ	*S,S*,Λ
A2780	3.5 ± 1.3	4.8 ± 0.5
A2780cp	7.3 ± 2.1	4.7 ± 2.0
A2780adr	6.5 ± 3.2	4.3 ± 2.1

**Figure 7:**
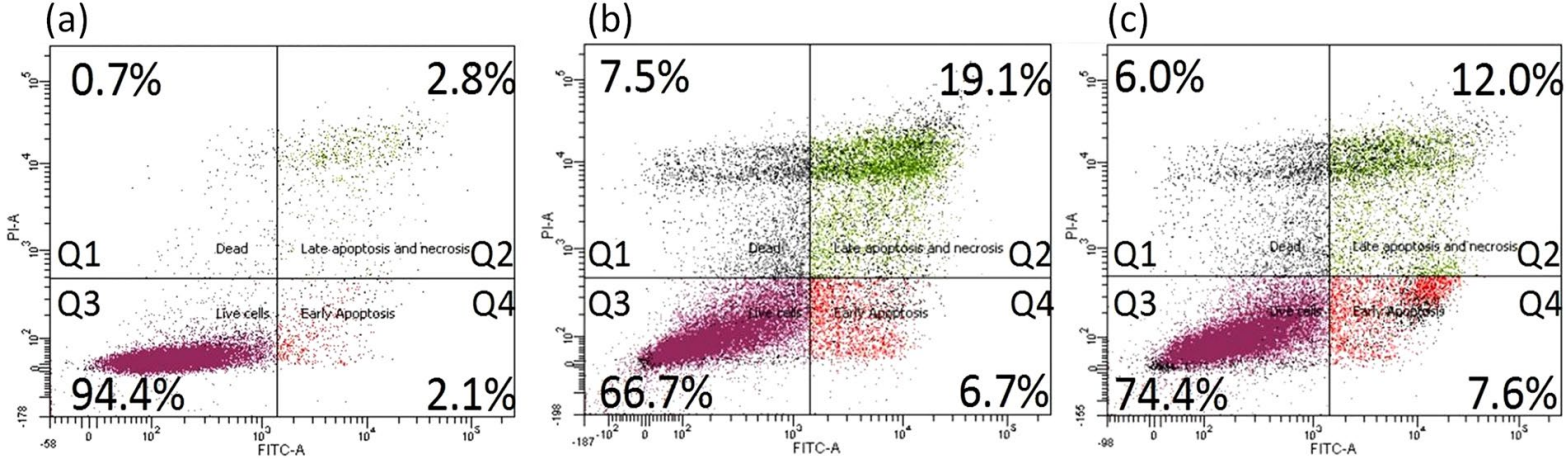
Effect of L^1^Ti(O*i*Pr)_2_ (8 *μ*M) ((**a**) control; (**b**) RR isomer; (**c**) SS isomer) on apoptosis (annexin V)/necrosis (propidium iodide) in human ovarian A2780 cells after 24 h of exposure using flow cytometry. Q1- necrotic (dead) cells; Q2- late apoptotic cells; Q3- viable cells; Q4- early apoptotic cells.

The induction of apoptosis vs. necrosis by the two enantiomers of L^1^Ti(O*i*Pr)_2_ was investigated *in vitro* by double staining A2780 cells with annexin V-FITC and propidium iodide using flow cytometry. The cells were exposed to 8 *μ*M (2xIC_50_) of racemic (*R,R*,Δ + *S,S*,Λ)-, *R,R*,Δ- or *S,S*,Λ-L^1^Ti(O*i*Pr)_2_ isomers for 24 hours. The distribution of the populations between early and late apoptosis and necrosis is depicted in Fig. 7. In correlation with the cytotoxicity behavior on all tested cell lines, similar responses were observed for both enantiomers, showing similar induction of apoptosis within 24 hours. The racemic mixture had a negligible effect on the A2780 cells as anticipated by its inactivity, providing a distribution highly resembling that obtained in the control experiment (Supplementary Fig. S4).

## Conclusions

Herein we presented new chiral derivatives of anticancer salan Ti(IV) complexes based on the chiral cyclohexyl moiety. The most stable derivative of the examined compounds, with *ortho*-bromination on the aromatic rings and a tertiary amino bridge, exhibited no biological activity for all isomers. This may be explained by the additive effect of the steric groups, evincing that more stable is not always more active^38,40,43^. Inspecting the hydrolytic behavior of the least stable NH complex, it is evident that polynuclear complexes are obtained in water. Interestingly, the first product appears to be a dimer, where the cluster nuclearity increases with time to yield a tetranuclear species, that even after 30 days in water does not decompose significantly to give titanium dioxide. The pure enantiomers of this complex showed a marked cytotoxicity on all lines tested; nevertheless, different reactivity of the racemic mixture supports the notion that the polynuclear hydrolysis products are the cellular active species^37,41–44^.

Comparing the behavior of the two active enantiomers in various aspects reveal no apparent difference, implying a similar mechanism^35,36,55^. Both are similarly active on all lines tested, and similarly induce apoptosis. It is thus evident that the Ti(IV) complexes perform through a different mechanism than that of cisplatin, adriamycin, or related drugs, providing them a potential advantage in the clinic. Additionally, similar reactivity of the two enantiomers may imply that tedious separation of enantiomers of chiral anticancer Ti(IV) complexes may be unnecessary, which is certainly another advantage. Nevertheless, identifying the biological target, if not necessarily chiral, and the mode of operation of these complexes remains enigmatic, and certainly merit additional mechanistic studies, currently underway in our laboratory.

## Methods

Ligands L^1–3^H_2_ and bis(isopropoxo) titanium(IV) complexes L^1–3^Ti(O*i*Pr)_2_ were synthesized according to published procedures^37,38,48^. All bis(isopropoxo) complexes were obtained in quantitative yields. Paraformaldehyde (97%), NaBH_4_ (97%), 1,2-*trans*-cyclohexanediamine (99%), and all substituted phenol and salicylaldehyde compounds (>96%) were purchased from Aldrich Chemical Company Inc., Fluka Riedel-DeHaen, Strem Chemicals Inc. or Alfa Aesar. Titanium tetra(isopropoxide) (97%) was purchased from Sigma Aldrich Chemical Company (Merck group). All solvents were dried over aluminum column on an MBraun drying system SPS-800. All experiments requiring dry atmosphere were performed in an M. Braun or LC-technologies dry-box or under nitrogen atmosphere using Schlenck line technique. NMR spectroscopic data were recorded with an AMX-400 MHz or AMX-500 MHz Bruker spectrometer. X-ray diffraction data were obtained with Bruker Smart Apex diffractometer. High resultion electrospray ionisation mass spectrometry were performed in the microanalytical laboratory in our institute. Specific optical rotation measurements were performed by Autopol I Automatic Polarimeter from Rudolph Research analytical and were calculated as the average of five measurements.

Cytotoxicity was measured on human colon HT-29 cancer cells (purchased from ATCC Inc.), human ovarian carcinoma A2780, human ovarian cisplatin-resistant carcinoma A2780cp, and human ovarian adriamycin-resistant A2780adr carcinoma cancer cell lines (purchased from ECACC Inc.) using the MTT assay as previously described^54^. Each measurement was repeated at least 3 × 3 times, namely, three repeats per plate, all repeated three times on different days (9 repeats altogether). Relative IC_50_ values with standard error of means were determined by a nonlinear regression of a variable slope (four parameters) model by Graph Pad Prism5.0 program. Kinetic hydrolysis studies by NMR were performed at RT as previously described^25^, using ca. 3.5 mM of the complex solution in THF-*d*_8_ and adding >1000 equiv. of D_2_O to give a final solution of 1:9 D_2_O/THF-*d*_8_. The ${{\rm{t}}}_{\frac{1}{2}}$ value is based on a pseudo first order fit for each compound. The results were verified by including *p*-dinitrobenzene (Sigma Aldrich Chemical Company Inc.) as an internal standard.

Apoptosis was measured using MEBCYTO apoptosis kit (annexin V-FITC kit, MBL). Cells were cultured in 6-well plates at density of 100,000 cells per well and allowed to attach overnight. The next day, the complex was added at 2xIC_50_ (8 *μ*M) concentration and was incubated for 24 h. All procedures were conducted according to the manufacturers instructions. The samples were analyzed by flow cytometry (Becton-Dickinson Excallibar Fluorescence Activated Cell Sorter).

### *Rac*-L^1^H_2_

3-Bromo-2-hydroxy-benzaldehyde (0.90 g, 4.5 mmol) and *trans*-1,2-cyclohexanediamine (0.3 mL, 2.2 mmol) in methanol (40 mL) were heated to reflux for 2 hours. The reaction was cooled to 0 °C and ca. 15 equivalents of NaBH_4_ (1.2 g, 33 mmol) were added. The precipitate was filtered and washed with cold methanol to give the desired product in 73% yield. ESI-HRMS (C_20_H_24_Br_2_N_2_O_2_ + H)^+^ m/z Calc.: 485.026 [M^+^] Found: 485.02690. ^1^H NMR (400 MHz, CDCl_3_): *δ* = 7.42 (d, *J* = 8.1 Hz, 1H; Ar), 7.01 (d, *J* = 7.6 Hz, 1H; Ar), 6.71 (t, *J* = 7.6 Hz, 1H; Ar), 4.07 (d, *J* = 13.6 Hz, 1H; CH_2_), 3.92 (d, *J* = 13.6 Hz, 1H; CH_2_), 2.54 (m, 1H; cy), 2.13 (m, 1H; cy), 1.74 (m, 1H; cy), 1.28 (m, 2H; cy) ppm. ^13^C NMR (125 MHz, CDCl_3_): *δ* = 154.6, 132.1, 127.2, 124.5, 120.0, 110.8, 59.7, 49.6, 30.7, 24.0 ppm.

***R,R****,*Δ- (68%) and ***S,S****,*Λ-L^1^H_2_ (69%) were prepared similarly to *rac*-L^1^H_**2**_ from the optically pure *trans*-1,2-cyclohexanediamine. ESI-HRMS (C_20_H_24_Br_2_N_2_O_2_ + H)^+^ m/z Calc.: 485.026 [M^+^]; *R,R*,Δ-L^1^H_2_ found 485.02475, *S,S*,Λ-L^1^H_2_ found 485.03051. Optical rotation for *R,R*,Δ-L^1^H_2_: [*α*]_D_^26^ = −35 ± 1°, for *S,S*,Λ-L^1^H_2_: [*α*]_D_^25^ = 28 ± 2° (for both: c = 3 mg/mL CHCl_3_).

### *Rac*-L^2^H_2_

5-Nitro-2-hydroxy-benzaldehyde (0.80 g, 4.8 mmol) and *trans*-1,2-cyclohexanediamine (0.3 mL, 2.4 mmol) in methanol (40 mL) were heated to reflux for 2 hours. The reaction was cooled to 0 °C and ca. 15 equivalents of NaBH_4_ (1.4 g, 36 mmol) were added. The precipitate was filtered and washed with cold methanol to give the desired product in 40% yield. ESI-HRMS (C_2__0_H_24_N_4_O_6_ + H)^+^ m/z Calc.: 417.177 [M^+^] Found: 417.17564. ^1^H NMR (400 MHz, DMSO): *δ* = 8.09 (s, 1H; Ar), 7.90 (dd, *J* = 4.6 Hz, 1H; Ar), 6.46 (s, 1H; Ar), 3.92 (d, *J* = 13.1 Hz, 1H; CH_2_), 3.77 (d, *J* = 13.2 Hz, 1H; CH_2_), 2.66 (m, 1H; cy), 2.07 (m, 1H; cy), 1.70 (m, 1H; cy), 1.23 (m, 2H; cy) ppm. ^13^C NMR (125 MHz, DMSO): *δ* = 132.2, 128.2, 127.0, 126.2, 125.6, 118.7, 58.9, 46.0, 29.5, 24.7 ppm.

***R,R****,*Δ- (56%) and ***S,S****,*Λ**-L**^**2**^**H**_**2**_ (66%) were prepared similarly to *rac*-L^2^H_2_ from optically pure *trans*-1,2-cyclohexanediamine. ESI-HRMS (C_20_H_24_N_4_O_6_ + H)^+^ m/z Calc.: 417.177 [M^+^]; *R,R*,Δ-L^1^H_2_ found 417.17668, *S,S*,Λ-L^1^H_2_ found 417.17773. Optical rotation for *R,R*,Δ-L^2^H_2_: [*α*]_D_^24^ = −19 ± 1°, for *S,S*,Λ-L^2^H_2_ [*α*]_D_^30^ = 29 ± 1° (for both: c = 3 mg/mL DMSO).

### *Rac*-L^3^H_2_

*Rac*-L^1^H_2_ (0.3 gr, 0.6 mmol) was dissolved in a mixture of acetonitrile and acetic acid (10 ml) and formaldehyde (15 mL, 0.41 mol) was added. The reaction was stirred at room temperature for ca. 2 hours to give a solid precipitate, and then cooled to 0 °C. Consequently, ca. 30 equivalents of NaBH_4_ (0.7 gr, 18 mmol) were added and the reaction was allowed to stand at room temperature overnight. NaOH 2M was added until pH 10 was reached. The precipitate was filtered and washed with water giving the desired product in 33% yield. ESI-HRMS (C_22_H_28_Br_2_N_2_O_2_ + H)^+^ m/z Calc.: 513.058 [M^+^] Found: 513.06056. ^1^H NMR (400 MHz, CDCl_3_): *δ* = 7.43 (d, *J* = 7.7 Hz, 1H; Ar), 6.95 (d, *J* = 7.4 Hz, 1H; Ar), 6.67 (t, *J* = 7.4 Hz, 1H; Ar), 3.86 (d, *J* = 13.7 Hz, 1H; CH_2_), 3.63 (d, *J* = 12.9 Hz, 1H; CH_2_), 2.70 (m, 1H; cy), 2.24 (s, 3 H; CH_3_), 2.01 (m, 1H; cy), 1.82 (m, 1H; cy), 1.21 (m, 2H; cy) ppm. ^13^C NMR (125 MHz, CDCl_3_): *δ* = 154.7, 132.5, 128.2, 123.9, 119.9, 110.9, 61.8, 56.5, 35.8, 25.2, 22.9 ppm.

***R,R****,*Δ- (31%) and ***S,S****,*Λ**-L**^**3**^**H**_**2**_ (43%) were prepared similarly to *rac*-L^3^H_2_ from optically pure L^1^H_2_. ESI-HRMS (C_22_H_28_Br_2_N_2_O_2_ + H)^+^ m/z Calc.: 513.058 [M^+^]; *R,R*,Δ-L^3^H_2_ found 513.06862, *S,S*,Λ -L^3^H_2_ found 513.07273. Optical rotation for *R,R*,Δ-L^3^H_2_: [*α*]_D_^33^ = 29 ± 2°, for *S,S*,Λ-L^3^H_2_: [*α*]_D_^29^ = −26 ± 1° (for both: c = 3 mg/mL CHCl_3_).

### *Rac*-L^1^Ti(O*i*Pr)_2_

Ti(O*i*Pr)_4_ (0.044 g, 0.15 mmol) was reacted with *rac*-L^1^H_2_ (0.075 g, 0.15 mmol) in dry THF at room temperature for 2 hours. After evaporation, the crude product was obtained in a >95% purity in quantitative yield. ESI-HRMS (C_26_H_36_Br_2_N_2_O_4_Ti + H)^+^ m/z Calc.: 649.058 [M^+^] Found: 649.05792. ^1^H NMR (400 MHz, CDCl_3_): *δ* = 7.45 (d, *J* = 7.8 Hz, 1H; Ar), 6.89 (d, *J* = 7.4 Hz, 1H; Ar), 6.52 (t, *J* = 7.6 Hz, 1H; Ar), 5.09 (sept, *J* = 6.1 Hz, 1H; CH), 4.87 (d, *J* = 14.2 Hz, 1H; CH_2_), 3.89 (d, *J* = 14.2 Hz, 1H; CH_2_), 2.22 (m, 1H; cy), 1.86 (m, 1H; cy), 1.65 (m, 1H; cy), 1.23 (m, 6H; CH_3_), 0.99 (m, 1H; cy), 0.85 (m, 1H; cy) ppm. ^13^C NMR (125 MHz, THF-*d*_8_): *δ* = 158.7, 131.4, 128.3, 124.3, 117.2, 112.3, 77.0, 62.7, 57.8, 48.9, 28.4, 25.4, 25.3 ppm.

***R,R****,*Δ- and ***S,S****,*Λ**-**L^1^**Ti(O*i*Pr**)_2_ were prepared similarly to *rac*-L^1^Ti(O*i*Pr)_2_ from optically pure L^1^H_2_. ESI-HRMS (C_26_H_36_Br_2_N_2_O_4_Ti + H)^+^ m/z Calc.: 649.058 [M^+^]; *R,R*,Δ-L^1^Ti(O*i*Pr)_2_ found 649.05792, *S,S*,Λ-L^1^Ti(O*i*Pr)_2_ found 649.05792. Optical rotation for *R,R*,Δ-L^1^Ti(O*i*Pr)_2_: [*α*]_D_^24^ = −75 ± 6°, for *S,S*,Λ-L^1^Ti(O*i*Pr)_2_: [*α*]_D_^27^ = 88 ± 6° (for both: c = 0.6 mg/mL CHCl_3_).

### Crystal data for Rac-L^1^Ti(OiPr)_2_

C_26_H_36_Br_2_N_2_O_4_Ti, Mr = 648.29, monoclinic, *a* = 13.052(1) Å, *b* = 15.173(1) Å, *c* = 13.742(1) Å, *β* = 93.513(2)°, *V* = 2716.3(4) Å^3^, *T* = 173(1) K, space group *P2*_1/n_, *Z* = 4, *μ* (Mo-K_*α*_) = 3.291 mm^−1^, 31165 reflections measured, 6490 unique (Rint = 0.0482). *R*(*F*^2^_o_) for [*I* > 2 *σ*(I)] = 0.0429, *R*_w_ for [*I* > 2 *σ*(*I*)] = 0.0861.

### *Rac*-L^2^Ti(O*i*Pr)_2_

Ti(O*i*Pr)_4_ (0.058 g, 0.20 mmol) was reacted with *rac*-L^2^H_2_ (0.085 g, 0.20 mmol) in dry THF at room temperature overnight. After evaporation, the crude product was obtained in a >95% purity in quantitative yield. ESI-HRMS (C_26_H_36_N_4_O_8_Ti + H)^+^ m/z Calc.: 581.209 [M^+^] Found: 581.20880. ^1^H NMR (400 MHz, DMSO): *δ* = 8.08 (d, *J* = 2.1 Hz, 1H; Ar), 8.00 (dd, *J* = 7.2 Hz, 1H; Ar), 6.64 (d, *J* = 7.2 Hz, 1H; Ar), 4.79 (sept, *J* = 4.88 Hz, 1H; CH), 4.40 (d, *J* = 13.2 Hz, 1H; CH_2_), 4.02 (d, *J* = 10.6 Hz, 1H; CH_2_), 2.14 (m, 1H; cy), 2.03 (m, 1H; cy), 1.55 (m, 1H; cy), 1.14 (m, 6H; CH_3_), 1.04 (m, 1H; cy), 0.81 (m, 1H; cy) ppm. ^13^C NMR (125 MHz, DMSO): *δ* = 169.5, 137.5, 126.3, 125.4, 124.0, 118.5, 77.6, 67.5, 62.4, 58.5, 26.0, 25.6, 24.5 ppm.

***R,R****,*Δ- and ***S,S****,*Λ**-**L^2^**Ti(O*i*Pr**)_**2**_ were prepared similarly to *rac*-L^2^Ti(O*i*Pr)_2_ from optically pure L^2^H_2_. ESI-HRMS (C_26_H_36_N_4_O_8_Ti + H)^+^ m/z Calc.: 581.209 [M^+^]; *R,R*,Δ-L^2^Ti(O*i*Pr)_2_ found 581.20880, *S,S*,Λ-L^2^Ti(O*i*Pr)_2_ found 581.21200. Optical rotation for *R,R*,Δ-L^2^Ti(O*i*Pr)_2_: [*α*]_D_^25^ = −270 ± 10° (c = 0.1 mg/mL DMSO), for *S,S*,Λ- L^2^Ti(O*i*Pr)_2_: [*α*]_D_^19^ = 249 ± 2° (c = 0.1 mg/mL DMSO).

### *Rac*-L^3^Ti(O*i*Pr)_2_

Ti(O*i*Pr)_4_ (0.050 g, 0.17 mmol) was reacted with *rac*-L^3^H_2_ (0.090 g, 0.17 mmol) in dry THF at room temperature over weekend. After evaporation, the crude product was obtained in a >95% purity in quantitative yield. ESI-HRMS (C_28_H_40_Br_2_N_2_O_4_Ti + H)^+^ m/z Calc.: 677.089 [M^+^] Found: 677.08929. ^1^H NMR (400 MHz, CDCl_3_): *δ* = 7.46 (d, *J* = 6.2 Hz, 1H; Ar), 6.89 (d, *J* = 5.6 Hz, 1H; Ar), 6.54 (t, *J* = 6.1 Hz, 1H; Ar), 5.42 (sept, *J* = 4.96 Hz, 1H; CH), 4.71 (d, *J* = 10.9 Hz, 1H; CH_2_), 3.30 (d, *J* = 10.8 Hz, 1H; CH_2_), 2.55 (m, 1H; cy), 2.37 (s, 3H; CH_3_), 1.66 (m, 1H; cy), 1.56 (m, 1H; cy), 1.27 (m, 6H; CH_3_), 1.06 (m, 1H; cy), 0.74 (m, 1H; cy) ppm. ^13^C NMR (125 MHz, CDCl_3_): *δ* = 158.4, 132.8, 128.5, 127.2, 118.1, 112.7, 78.9, 68.0, 61.0, 57.8, 42.1, 25.6, 22.1, 19.2 ppm.

***R,R****,*Δ- and ***S,S****,*Λ**-L**^**3**^**Ti(O*i*Pr**)_**2**_ were prepared similarly to *rac*-L^3^Ti(O*i*Pr)_2_ from optically pure L^3^H_2_. ESI-HRMS (C_28_H_40_Br_2_N_2_O_4_Ti + H)^+^ m/z Calc.: 677.089 [M^+^], *R,R*,Δ-L^3^Ti(O*i*Pr)_2_ found 677.08929; (C_28_H_40_Br_2_N_2_O_4_Ti + Na)^+^ m/z Calc.: 699.071 [M^+^], *S,S*,Λ-L^3^Ti(O*i*Pr)_2_ found 699.07039. Optical rotation for *R,R*,Δ-L^3^Ti(O*i*Pr)_2_: [*α*]_D_^27^ = −350 ± 10° (c = 0.1 mg/mL CHCl_3_), for *S,S*,Λ-L^3^Ti(O*i*Pr)_2_: [*α*]_D_^24^ = 350 ± 20° (c = 0.2 mg/mL CHCl_3_).

### Crystal data for *Rac*-L^3^Ti(O*i*Pr)_2_

C_28_H_40_Br_2_N_2_O_4_Ti, *M*r = 676.34, monoclinic, *a* = 16.718(4) Å, *b* = 10.090(2) Å, *c* = 18.406(4) Å, *β* = 110.739(3)°, *V* = 2903.7(11) Å^3^, *T* = 173(1) K, space group *P*2_1/c_, *Z* = 4, *μ* (Mo-K_*α*_) = 3.082 mm^−1^, 29963 reflections measured, 6286 unique (Rint = 0.0664). *R*(*F*^2^_o_) for [*I* > 2 *σ*(*I*)] = 0.0825, R_w_ for [*I* > 2 *σ*(*I*)] = 0.1623.

### Crystal data for *S,S*,Λ-L^3^Ti(O*i*Pr)_2_

C_28_H_40_Br_2_N_2_O_4_Ti, *M*r = 676.34, monoclinic, *a* = 10.0049(6) Å, *b* = 14.4108(9) Å, *c* = 10.2129(6) Å, *β* = 96.098(1)°, *V* = 1464.2(2) Å^3^, T = 173(1) K, space group *P*2_1_, *Z* = 2, *μ*(Mo-K^*α*^) = 3.057 mm^−1^, 16766 reflections measured, 6789 unique (Rint = 0.0231). *R*(*F*^2^_o_) for [*I* > 2 *σ*(*I*)] = 0.0259, *R*_w_ for [*I* > 2 *σ*(*I*)] = 0.0554.

The hydrolysis product **Ti**_**4**_**(***μ*_**-O)4(S,S-L**_^**1**^**)**_**4**_ was obtained by dissolving ca. 20 mg of *S,S*,Λ-L^1^Ti(O*i*Pr)_2_ in THF (6 mL) and adding >10,000 equivalents of H_2_O. The reaction was mixed for 30 days. The product was crystallized from diethyl ether. The structure contains disordered water molecules, for which H atoms were not detected.

### Crystal data for Ti_4_(*μ*-O)_4_(S,S-L^1^)_4_

C_80_H_88_Br_8_N_8_O_17.33_Ti_4_, *M*r = 2269.80, rhombohedral, *a* = 26.4920 (9) Å, *c* = 70.771 (3) Å, *V* = 43015(3) Å^3^, *T* = 173(1) K, space group *R* $\overline{3}$ *c*, Z = 18, *μ* (Mo-K_*α*_) = 3.730 mm^−1^, 159243 reflections measured, 11525 unique (Rint = 0.1489). *R*(*F*^2^_o_) for [*I* > 2 *σ*(*I*)] = 0.0660, R_w_ for [*I* > 2 *σ*(*I*)] = 0.1270.

CCDC 1817336–1817339 contain the supplementary crystallographic data for this paper. These data can be obtained free of charge from The Cambridge Crystallographic Data Centre via https://www.ccdc.cam.ac.uk/structures/.

## Electronic supplementary material

Supplementary Information
