# Supplementary material for: Synthesis of Pure Enantiomers of Titanium(IV) Complexes with Chiral Diaminobis(phenolato) Ligands and Their Biological Reactivity
Source: Sci Rep. 2018 Jun 26;8:9705. doi: 10.1038/s41598-018-27735-0 (PMC6018713; doi:10.1038/s41598-018-27735-0)
Supplement: Supplementary file 1 — Supplementary Information [file 41598_2018_27735_MOESM1_ESM.docx]

**Synthesis of Pure Enantiomers of Titanium(IV) Complexes with Chiral Diaminobis(phenolato) Ligands and Their Biological Reactivity**

Maya Miller and Edit Y. Tshuva*

*Institute of Chemistry, The Hebrew University of Jerusalem, Jerusalem 9190401, Israel. Email: edit.tshuva@mail.huji.ac.il*

**Supplementary Information**

**Table S1**. Selected bond lengths [Å] and angles [°] for *rac*-L^1^Ti(O*i*Pr)_2_, *rac*-L^3^Ti(O*i*Pr)_2_ and *S,S,Λ*-L^3^Ti(O*i*Pr)_2_.

| *Rac*-L^1^Ti(O*i*Pr)_2_ | | | |
| --- | --- | --- | --- |
| Lengths |  |  |  |
| O(1)–Ti | 1.950(2) | O(4)–Ti | 1.785(2) |
| O(2)–Ti | 1.930(2) | N(2)–Ti | 2.268(2) |
| O(3)–Ti | 1.791(2) | N(1)–Ti | 2.250(2) |
| Angles |  |  |  |
| O(4)–Ti–O(3) | 105.92(1) | O(2)–Ti–N(1) | 86.49(8) |
| O(4)–Ti–O(2) | 96.90(9) | O(1)–Ti–N(1) | 80.50(8) |
| O(3)–Ti–O(2) | 95.88(9) | O(4)–Ti–N(2) | 91.90(9) |
| O(4)–Ti–O(1) | 92.25(9) | O(3)–Ti–N(2) | 162.00(9) |
| O(3)–Ti–O(1) | 99.13(9) | O(2)–Ti–N(2) | 79.18(8) |
| O(2)–Ti–O(1) | 159.56(8) | O(1)–Ti–N(2) | 82.29(8) |
| O(4)–Ti–N(1) | 165.97(9) | N(1)–Ti–N(2) | 75.31(8) |
| O(3)–Ti–N(1) | 87.18(9) |  |  |
| *Rac*-L^3^Ti(O*i*Pr)_2_ | | | |
| Lengths |  |  |  |
| O(4)–Ti | 1.802(4) | O(1)–Ti | 1.930(4) |
| O(3)–Ti | 1.800(4) | N(2)–Ti | 2.357(4 |
| O(2)–Ti | 1.927(4) | N(1)–Ti | 2.324(4) |
| Angles |  |  |  |
| O(3)–Ti–O(4) | 106.77(2) | O(2)–Ti–N(1) | 88.03(2) |
| O(3)–Ti–O(2) | 96.73(2) | O(1)–Ti–N(1) | 81.23(2) |
| O(4)–Ti–O(2) | 92.01(2) | O(3)–Ti–N(2) | 89.81(2) |
| O(3)–Ti–O(1) | 91.04(2) | O(4)–Ti–N(2) | 162.59(2) |
| O(4)–Ti–O(1) | 96.94(2) | O(2)–Ti–N(2) | 80.53(2) |
| O(2)–Ti–O(1) | 165.95(2) | O(1)–Ti–N(2) | 87.84(2) |
| O(3)–Ti–N(1) | 162.60(2) | N(1)–Ti–N(2) | 74.42(2) |
| O(4)–Ti–N(1) | 89.71(2) |  |  |
| *S,S,Λ*-L^3^Ti(O*i*Pr)_2_ | | | |
| Lengths |  |  |  |
| O(1)–Ti | 1.914(2) | O(4)–Ti | 1.820(2) |
| O(2)–Ti | 1.918(2) | N(2)–Ti | 2.347(2) |
| O(3)–Ti | 1.817(2) | N(1)–Ti | 2.360(2) |
| Angles |  |  |  |
| O(3)–Ti–O(4) | 106.78(8) | O(1)–Ti–N(2) | 90.75(7) |
| O(3)–Ti–O(1) | 92.03(7) | O(2)–Ti–N(2) | 81.48(7) |
| O(4)–Ti–O(1) | 95.28(7) | O(3)–Ti–N(1) | 160.89(8) |
| O(3)–Ti–O(2) | 95.23(7) | O(4)–Ti–N(1) | 91.60(8) |
| O(4)–Ti–O(2) | 90.27(8) | O(1)–Ti–N(1) | 80.88(7) |
| O(1)–Ti–O(2) | 169.20(7) | O(2)–Ti–N(1) | 89.72(7) |
| O(3)–Ti–N(2) | 88.53(8) | N(2)–Ti–N(1) | 73.94(7) |
| O(4)–Ti–N(2) | 163.28(8) |  |  |

Figure S1: Aromatic rigion of the ^1^H NMR spectra for the hydrolysis product of *S*,*S*,*Λ*-L^1^Ti(O*i*Pr)_2_ in THF-*d*_8_, 24 h following addition of D_2_O

Figure S2: Aromatic rigion of the ^1^H NMR spectra for the hydrolysis product of *Rac*-L^1^Ti(O*i*Pr)_2_ in THF-*d*_8_, 24 h following addition of D_2_O

**Table S2**. Selected bond lengths [Å] and angles [°] for Ti_4_(µ-O)_4_(*S*,*S*-L^1^)_4_

| Ti_4_(µ-O)_4_(S,S-L^1^)_4_ | | | |
| --- | --- | --- | --- |
| Lengths |  | Lengths |  |
| N(1)–Ti(1) | 2.346(4) | O(3)–Ti(1) | 1.837(2) |
| N(2)–Ti(1) | 2.269(4) | O(4)–Ti(1) | 1.809(3) |
| N(3)–Ti(2) | 2.294(4) | O(4)–Ti(2) | 1.904(3) |
| N(4)–Ti(2) | 2.215(4) | O(5)–Ti(2) | 1.886(3) |
| O(1)–Ti(1) | 1.910(3) | O(6)–Ti(2) | 1.911(3) |
| O(2)–Ti(1) | 1.968(3) | O(7)–Ti(2)'^a^ | 1.820(1) |
| O(3)–Ti(1)'^a^ | 1.837(2) | O(7)–Ti(2) | 1.820(1) |
| Angles |  | Angles |  |
| O(4)–Ti(1)–O(3) | 97.2(2) | O(7)–Ti(2)–O(5) | 101.1(1) |
| O(4)–Ti(1)–O(1) | 101.2(2) | O(7)–Ti(2)–O(4) | 92.7 (2) |
| O(3)–Ti(1)–O(1) | 99.3(2) | O(5)–Ti(2)–O(4) | 100.2(2) |
| O(4)–Ti(1)–O(2) | 93.8(2) | O(7)–Ti(2)–O(6) | 94.6(2) |
| O(3)–Ti(1)–O(2) | 161.4(1) | O(5)–Ti(2)–O(6) | 94.1(2) |
| O(1)–Ti(1)–O(2) | 93.2(2) | O(4)–Ti(2)–O(6) | 162.4(2) |
| O(4)–Ti(1)–N(2) | 102.4(2) | O(7)–Ti(2)–N(4) | 101.9(1) |
| O(3)–Ti(1)–N(2) | 82.3(1) | O(5)–Ti(2)–N(4) | 156.6(2) |
| O(1)–Ti(1)–N(2) | 156.0(2) | O(4)–Ti(2)–N(4) | 83.1(2) |
| O(2)–Ti(1)–N(2) | 80.7(2) | O(6)–Ti(2)–N(4) | 79.7(2) |
| O(4)–Ti(1)–N(1) | 174.6(2) | O(7)–Ti(2)–N(3) | 176.3(1) |
| O(3)–Ti(1)–N(1) | 83.7(2) | O(5)–Ti(2)–N(3) | 82.6(2) |
| O(1)–Ti(1)–N(1) | 84.0(2) | O(4)–Ti(2)–N(3) | 86.1(2) |
| O(2)–Ti(1)–N(1) | 84.0(2) | O(6)–Ti(2)–N(3) | 85.7(2) |
| N(2)–Ti(1)–N(1) | 72.4(2) | N(4)–Ti(2)–N(3) | 74.4(2) |

^a^' Symmetry transformations used to generate equivalent atoms: x-y+1/3,-y+2/3,-z+1/6


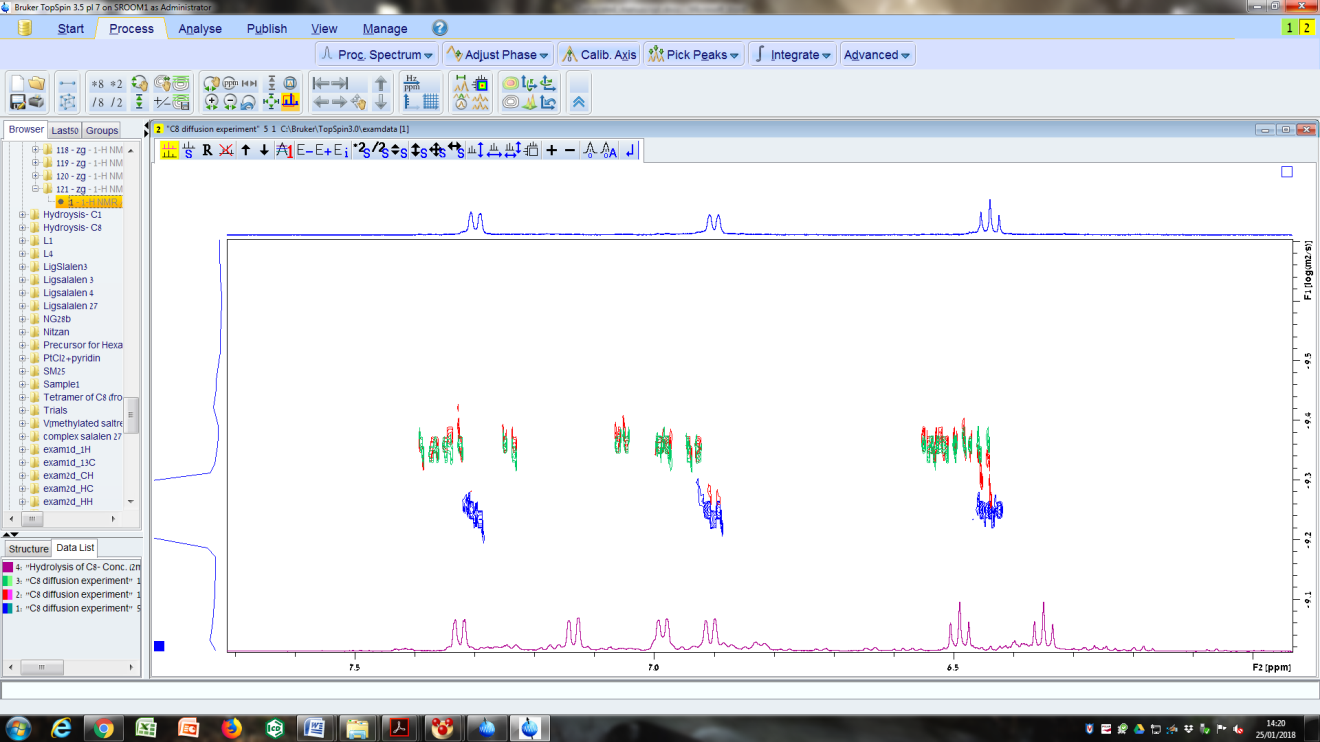


traces of presumably trinuclear species

Figure S3: Diffusion order spectroscopy, aromatic rigion of *S*,*S*,*Λ*-L^1^Ti(O*i*Pr)_2_ (blue) and its hydrolysis product in THF-*d*_8_, 24 h (red) and 48 h (green) following addition of D_2_O. ^1^H NMR spectra depicting *S*,*S*,*Λ*-L^1^Ti(O*i*Pr)_2_ (blue upper pannel), and its hydrolysis product in THF- *d*_8_, 48 h following addition of D_2_O (purple lower pannel).


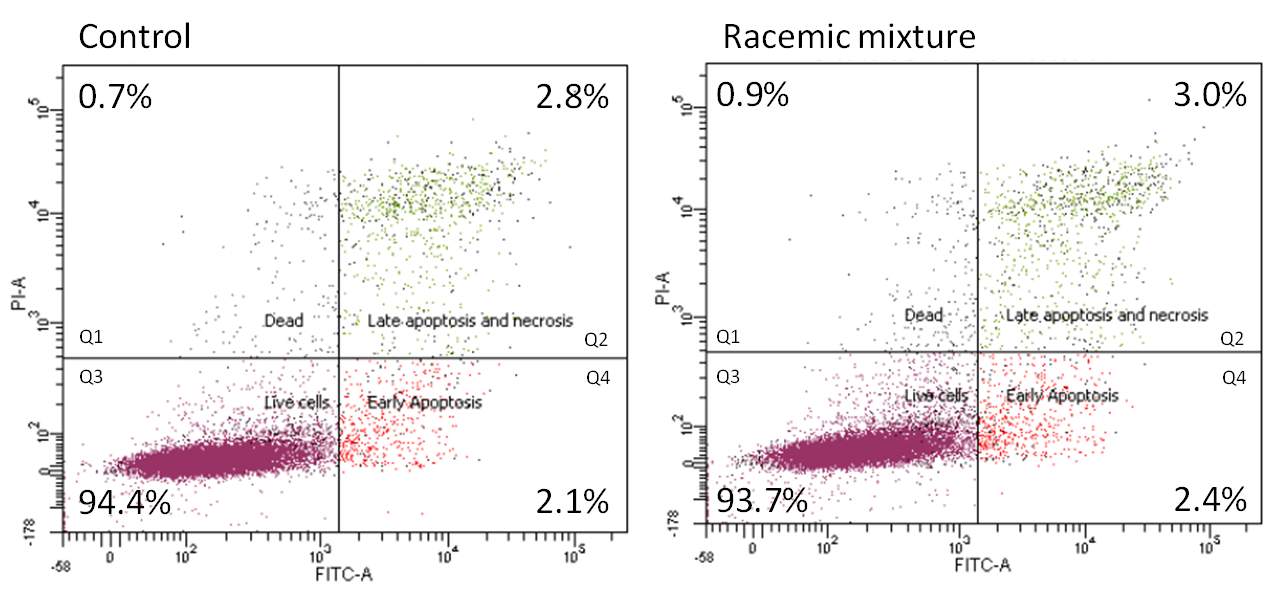


Figure S4: Effect of L^1^Ti(O*i*Pr)_2_ (8 µM) on apoptosis (annexin V) / necrosis (propidium iodide) in human ovarian A2780 cells after 24 h of exposure using flow cytometry. Q1- necrotic (dead) cells; Q2- late apoptotic cells; Q3- viable cells; Q4- early apoptotic cells.

Circular Dichroism (CD)

CD of the complexes were recorded on JASCO J-810 Spectrophotometer (JASCO, Japan) using supplied SpectraManager software. The temperature was kept constant at 20 ˚C using a temperature controlled water bath. Samples were prepared fresh from stock before each measurement. About 1 mg of the tested compound was dissolved in 200 µL in THF and used as stock solution. Consequently dilutions were made to obtain concentration ranging from 0.65 to 1.45 mM and were placed in a 1cm/mm quartz cell. The spectra were recorded in wavelength rang of λ = 220 to 500 nm, with 2 accumulations for each measurement and a data pitch of 0.1 nm. Background CD spectrum of the solvent was recorded and subtracted form each spectrum.

Figure S5: CD spectra of *R*,*R*,*∆*- and *S*,*S*,*Λ*- L^1^Ti(O*i*Pr)_2_

Figure S6: CD spectra of *R*,*R*,*∆*- and *S*,*S*,*Λ*- L^2^Ti(O*i*Pr)_2_

Figure S7: CD spectra of *R*,*R*,*∆*- and *S*,*S*,*Λ*- L^3^Ti(O*i*Pr)_2_
